# Activation of miR-500a-3p/CDK6 axis suppresses aerobic glycolysis and colorectal cancer progression

**DOI:** 10.1186/s12967-022-03308-8

**Published:** 2022-03-03

**Authors:** Yu Liu, Wentao Tang, Li Ren, Tianyu Liu, Meng Yang, Ye Wei, Yijiao Chen, Meiling Ji, Guosong Chen, Wenju Chang, Jianmin Xu

**Affiliations:** 1 Colorectal Cancer Center, Zhongshan Hospital, Fudan University, Shanghai, China; 2 Department of General Surgery, Zhongshan Hospital, Fudan University, Shanghai, China; 3 Shanghai Engineering Research Center of Colorectal Cancer Minimally Invasive, Shanghai, China; 4 National Clinical Research Center for Cancer, Tianjin Cancer Institute, Tianjin, China; 5 The State Key Laboratory of Molecular Engineering of Polymers and Department of Macromolecular Science, Shanghai, China

**Keywords:** Colorectal cancer, miR-500a-3p, CDK6, Glycolysis

## Abstract

**Background:**

Colorectal cancer (CRC) is one of the lethal cancers with a high mortality rate worldwide and understanding the mechanisms behind its progression is critical for improving patients’ prognosis and developing therapeutics. MiR-500a-3p has been demonstrated to be involved in the progression of several human cancers but its role in CRC remains unclear. The aim of this study is to uncover the expression pattern and mechanisms of action of miR-500a-3p during the CRC progression.

**Methods:**

The expression of miR-500a-3p and Cyclin-dependent kinases 6 (CDK6) in 134 CRC tissues were tested by quantitative PCR (qPCR) and immunohistochemistry staining (IHC), respectively. The effect of miR-500a-3p on cell proliferation was explored in vitro and in vivo. The glycolysis of CRC cells was determined by Mass Spectrometry and Seahorse XF 96 Extracellular Flux Analyzer. A dual-luciferase reporter assay was performed to validate the relationship between miR-500a-3p and CDK6.

**Results:**

miR-500a-3p was abnormally downregulated in CRC tissues and cell lines and was negatively associated with a worse prognosis. miR-500a-3p mimics impeded CRC cell proliferation in vitro and in vivo. miR-500a-3p inhibited glucose consumption, lactate and ATP production, and down-regulated the expression of hexokinase2 (HK2). In silico prediction combined with western blot and luciferase assay confirmed that CDK6 is a direct target of miR-500a-3p. Overexpression of CDK6 phenotypically rescued the inhibitory effect of miR-500a-3p on the proliferation and glycolysis of CRC cells.

**Conclusions:**

Our study revealed a potential tumor-suppressive role of miR-500a-3p in CRC, specifically targeting CDK6 and inhibiting cancer cell proliferation and aerobic glycolysis, which may provide new insights into novel prognostic biomarkers and therapeutic targets for CRC.

**Supplementary Information:**

The online version contains supplementary material available at 10.1186/s12967-022-03308-8.

## Background

Colorectal cancer (CRC) is the third most commonly diagnosed cancer in the world with the second-highest mortality rate [1]. The stage status of tumors often determine the prognosis of CRC patients. The 5-year survival rate of CRC patients ranges from greater than 90% in patients with stage I disease to slightly greater than 10% in patients with stage IV disease [2]. Therefore, understanding the mechanisms behind the CRC progression is the key to improving patients’ prognosis.

MicroRNAs (miRNAs) are a class of single-stranded non-coding RNAs with a length of 21–25 nucleotides. miRNAs regulate the expression of approximately 60% of the protein-coding genes [3]. Aerobic glycolysis, one of the central contributors to cancer progression [4], help cancer cell meet the energy demand, accumulate glycolytic intermediates for cancer biomass synthesis and create an acidic microenvironment [5]. Existing studies have proved that microRNAs play an important role in the progression of CRC by regulating key glycolytic genes directly or indirectly [6, 7]. For example, miR-34a-5p inhibited HK1-mediated glycolysis while the miR-122-PKM2 axis contributed to attenuated glycolysis in CRC [8, 9]. miR-500a is located within the p11 locus on the X chromosome and plays various roles in cancer. miR-500a is upregulated in prostate cancer and promotes cell proliferation and invasion [10]. miR-500a promotes cell stemness via different pathways in gastric and liver cancer [11, 12]. However, miR-500a suppresses cell proliferation, migration and invasion in non-small cell lung cancer [13]. According to the data of a cohort of 551 CRC patients derived from the cBioportal database, miR-500a has the most significant correlation with prognosis (with the lowest *P* value). miR-500a has two arms, miR-500a-5p and miR-500a-3p. miR-500a-5p is downregulated and acts as a tumor suppresser in CRC. miR-500a-5p may attenuate epithelial‑mesenchymal transition through targeting the transforming growth factor‑β signaling pathway [14] and suppress cell proliferation by targeting the p300/YY1/HDAC2 axis [15]. However, the role of miR-500a-3p has not been elucidated in CRC.

Cyclin-dependent kinases 6 (CDK6) is known as a classic cell cycle kinase that facilitates the proliferation of cells through the early G1 phase of the cell cycle. CDK6 participates in the process of cancer progression through the kinase-dependent or non-kinase-dependent function [16]. Recently, the role of CDK6 in metabolic regulation has been reported. Wang et al. found that CDK6 regulates the catalytic activity of two key enzymes in the glycolytic pathway, 6-phosphofructokinase and pyruvate kinase M2 [17]. Nevertheless, the role of CDK6 in regulating CRC metabolism remains to be revealed.

In the current study, we investigated the effects of miR-500a-3p on the cell proliferation and glycolysis in CRC using patient samples and cell lines and uncovered its potential link to CDK6.

## Results

### miR-500a-3p is down regulated in tumor tissue and its low expression predicts poor prognosis

**Fig. 1:**
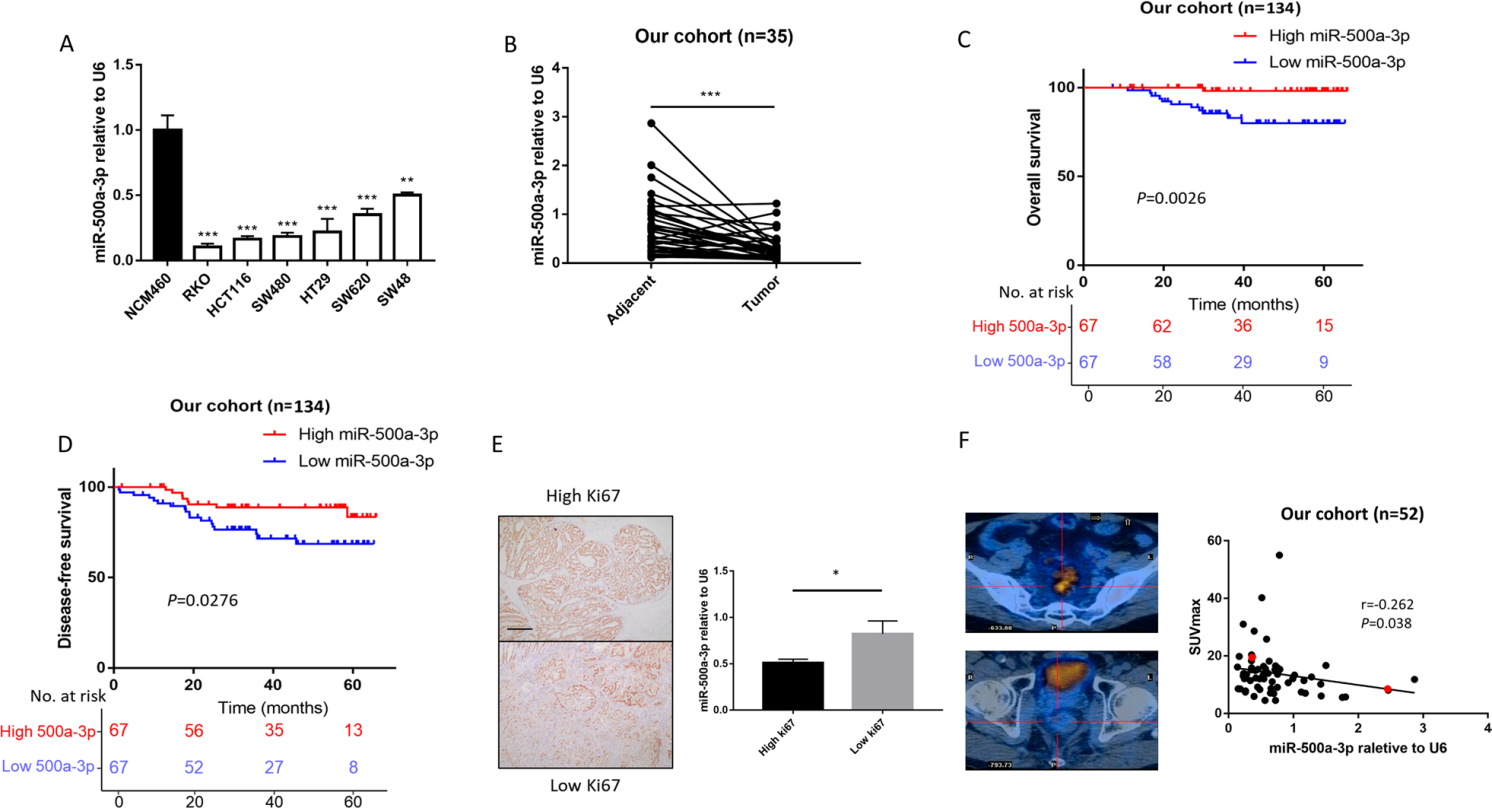
miR-500a-3p is down regulated in CRC and its low expression predicts poor prognosis. **A** The enrichment of miR-500a-3p was determined in normal human colon epithelial cell line NCM460 and six CRC cell lines by RT-qPCR. **B** The expression of miR-500a-3p was detected by qPCR in 20 pairs of CRC tissues. **C**, **D** The Kaplan–Meier survival curves of overall survival (**C**) and disease-free survival (**D**) in the cohort which contained 134 continuous CRC specimens. Patients were divided into two groups according to the expression of miR-500a-3p. Median expression levels were used as the cutoff. **E** Representative images of ki67 IHC staining (left) and quantification data (right) were shown. Scale bar, 100 μm. **F** The association between miR‐500a‐3p mRNA level and glucose uptake in 52 patients receiving PETCT preoperatively (Spearman’s correlation analysis). Glucose uptake was represented by Standard Uptake Value of PET-CT. The left pictures showed the PET-CT images of the patients indicated by the red dot. *P < 0.05, **P < 0.01, ***P < 0.001

According to the data of a cohort of 551 patients from the cBioportal database, we found that the expression of has-mir-500a was significantly associated with the prognosis of CRC patients (Additional file 1: Table S1) Patients who had low miR-500a expression had significantly poor overall survival (*P* = 0.008, Additional file 2: Fig. S1A) and progression-free survival (*P* < 0.001, Additional file 2: Fig. S1B). We firstly examined the miR-500a-3p expression by qPCR in CRC cell lines, including HCT116, HT29, RKO, SW620, SW48 and SW480, and normal human colon epithelial cell line NCM460. The expression level of miR-500a-3p was significantly down-regulated in CRC cells than that in NCM460 cells (Fig. 1A). In addition, it was significantly downregulated in CRC tissues compared to adjacent non-tumor tissues (Fig. 1B). Next, we examined miR-500a-3p expression in the cohort that contained 134 continuous CRC specimens. We divided the patients into low or high-expression groups by a cut-off of the median expression value. We found that the expression level of miR-500a-3p was negatively correlated with tumor size (*P* = 0.022) and differentiation (*P* = 0.043) (Table 1). Survival analysis showed that low miR-500a-3p expression was significantly correlated with poor overall survival (*P* = 0.0026, Fig. 1C) and disease-free survival (*P* = 0.0276, Fig. 1D). To further evaluate the prognostic value of miR-500a-3p, both univariate and multivariate analyses were performed. The results indicated that high miR-500a-3p expression is an independent protective factor for CRC [HR, 0.109; 95% confidence interval (CI), 0.014–0.875; *P* = 0.037]. (Additional file 1: Table S2). We also analyzed the correlation of miR-500a-3p expression with the biomarkers of cell proliferation (Ki-67) and glucose uptake (Standard Uptake Value, SUV) in CRC tissues. The expression of miR-500a-3p was significantly lower in the high Ki67 group than that in the low Ki67 group (Fig. 1E). Besides, in patients receiving PETCT preoperatively (n = 52), there was a negative correlation between the expression of miR-500a-3p and SUV with the *P* value of 0.038 and r value of -0.262 (Fig. 1F). Altogether, low expression level of miR-500a-3p predicts poor prognosis in CRC patients and it may suppress CRC by regulating tumor proliferation and glucose metabolism.

**Table 1:** Correlation of miR-500a-3p expression with clinicopathologic characteristics in colorectal cancer

Characteristics	miR-500a-3p expression		*P*
	High (n = 67)	Low (n = 67)	
Gender			0.587
Male	42 (62.7)	45 (67.2)	
Female	25 (37.3)	22 (32.8)	
Age, year	61.0 ± 13.0	63.6 ± 10.0	0.227
Location			0.089
Right side	18 (26.9)	10 (14.9)	
Left side	49 (73.1)	57 (85.1)	
Tumor size			0.022
< 5 cm	21 (31.3)	34 (50.7)	
≥ 5 cm	46 (68.7)	33 (49.3)	
Differentiation			0.043
Well/moderately	46 (68.7)	56 (83.6)	
Poor	21 (31.3)	11 (16.4)	
Depth of tumor			0.259
T1–T3	58 (86.6)	62 (92.5)	
T4	9 (13.4)	5 (7.5)	
Lymph node			0.119
Positive	31 (46.3)	40 (59.7)	
Negative	36 (53.7)	27 (40.3)	
Distant metastasis			0.825
Yes	55 (82.1)	54 (80.6)	
No	12 (17.9)	13 (19.4)	
TNM stage			0.226
I–II	28 (41.8)	35 (52.2)	
III–IV	39 (58.2)	32 (47.8)	

### miR-500a-3p suppresses CRC growth in vitro and in vivo

**Fig. 2:**
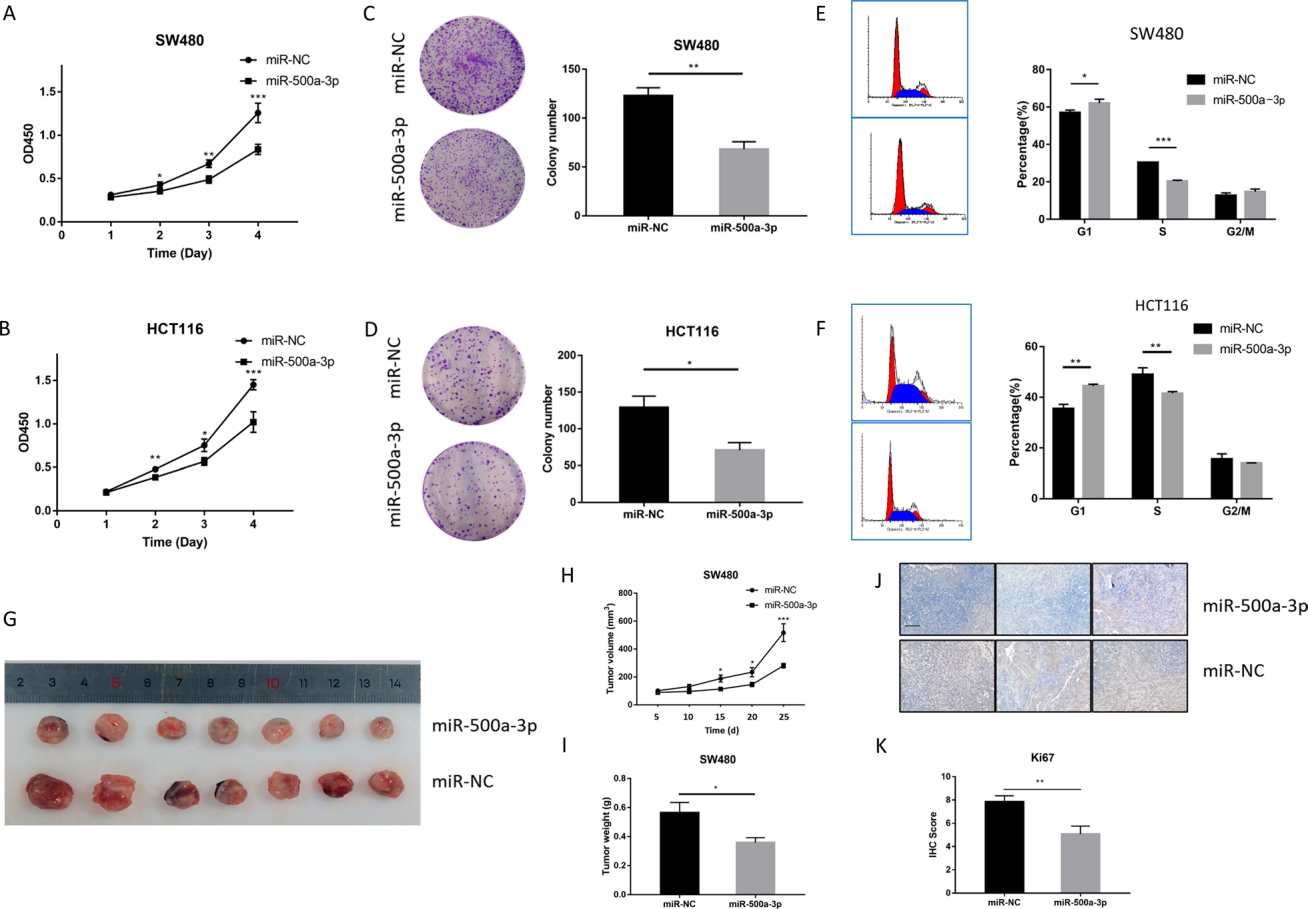
miR-500a-3p suppress CRC growth in vitro and in vivo. **A**, **B** CCK-8 assay showed the proliferation ability after transfection of miR-NC and miR-500a-3p in SW480 and HCT-116 cells. **C**, **D** The plate colony formation ability of SW480 and HCT-116 cells after transfection with miR-NC or miR-500a-3p mimics were measured by colony formation assay. Representative images (left) and quantification (right) were shown. **E**, **F** Flow cytometry showed the cell cycle in SW480 and HCT-116 cells. The data from **A** to **F** were presented as the means ± SD (n = 3). **G** Nude mice (n = 7) were subcutaneously injected with SW480 cells transfected with miR-NC or miR-500a-3p. Representative tumor diagrams in different groups were shown. **H**, **I** Volume and weight of xenograft tumor were measured in the miR-500a-3p and miR-NC group. Tumor volume was analyzed every 5d using the formula: volume = (length × width2)/2. **J**, **K** The subcutaneous tumors were stained with ki67. Scale bar, 100 μm. The quantification of ki67 IHC score in miR-500a-3p and miR-NC group were shown (n = 7). *P < 0.05, **P < 0.01, ***P < 0.001

HCT116/SW480 cells were transfected with miR-500a-3p mimics to investigate the function of miR-500a-3p in vitro. Transfection efficiencies were confirmed by RT-qPCR (Additional file 2: Fig. S2A). CCK-8 and colony formation assays indicated that CRC cell proliferation and colony formation were significantly inhibited by miR-500a-3p mimics (Fig. 2A–D). Next, fluorescence-activated cell sorting (FACS) analysis revealed an increase in G1-phase cells and a concomitant decrease in S-phase cells by miR-500a-3p (Fig. 2E, F). We also found that miR-500a-3p mimics did not promote apoptosis significantly (Additional file 2: Fig. S2B). Transwell assay showed that the miR-500a-3p mimics markedly inhibited CRC cell invasion (Additional file 2: Fig. S2C). We established xenograft tumor models with SW480 and HCT116 cells stably overexpressing miR-500a-3p (Additional file 2: Fig. S2D, E). The mean volume and weight of tumors were lower in the miR-500a-3p-overexpressing group than the vector group (Fig. 2G–I and Additional file 2: Fig. S2F–H). The protein expression of cell proliferation (Ki-67) in the xenograft tumors was lower in miR-500a-3p overexpression group than vector group (Fig. 2J). Altogether, these results indicated that miR-500a-3p suppresses CRC cell proliferation in vitro and in vivo.

### miR-500a-3p suppresses aerobic glycolysis in CRC

**Fig. 3:**
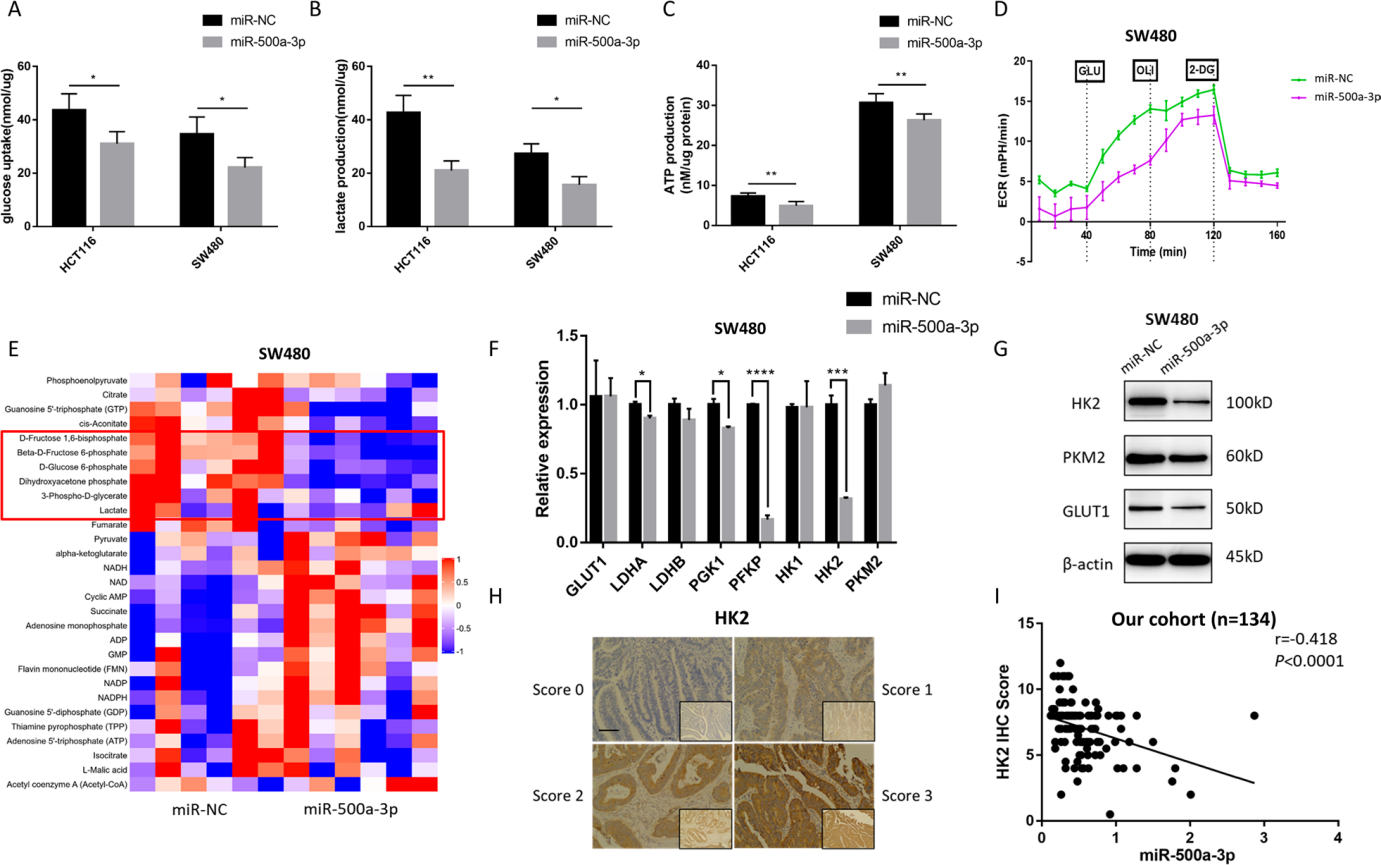
miR-500a-3p suppresses aerobic glycolysis in CRC. **A**–**C** The glucose uptake (**A**), lactate production (**B**), intracellular ATP (**C**) were measured in HCT116 and SW480 cells after cultured with FBS-free medium for 24 h using Glucose Uptake Colorimetric Assay kit, Lactate Assay Kit and ATP Colorimetric Assay kit. **D** The extracellular acidification rate (ECAR) of SW480 cells were analyzed by Seahorse XF96 Extracellular Flux Analyzer. The data from **A** to **D** were presented as the means ± SD (n = 3). **E** Heatmap showed the level of 29 metabolites related to energy metabolism in SW480 cells transfected with miR-NC and miR-500a-3p. 5 glycolytic metabolites whose levels were significantly different between two groups were marked in the red box. **F**, **G** The mRNA level of HK2, PFKP, PGK1 and LDHA (**F**) by qPCR and the protein level of GLUT1, HK2 and PKM2 (**G**) by western blot in SW480 cells transfected with miR-NC and miR-500a-3p. β-Actin served as control. **H** The specimens in the cohort which contained 134 continuous CRC patients were stained with HK2. Scale bar, 100 μm. **I** Association between miR-500a-3p mRNA level and HK2 IHC score were conducted in the cohort (n = 134). (Spearman’s correlation analysis) *P < 0.05, **P < 0.01, ***P < 0.001, ****P < 0.0001

The expression of miR-500a-3p in CRC patients is negatively correlated with glucose uptake, indicating that miR-500a-3p may have an inhibitory effect on glycolysis. Cellular glycolytic activity was measured. As a result, overexpression of miR-500a-3p significantly decreased glucose uptake (Fig. 3A), lactate production (Fig. 3B) and intracellular ATP levels (Fig. 3C) in both HCT116 and SW480 cells. The extracellular acidification rate (ECAR), measured by the Seahorse analyzer, was downregulated by miR-500a-3p (Fig. 3D). Furthermore, targeted metabolomics focused on the energy metabolism pathway was performed, and lower level of glycolytic metabolites like glucose 6-phosphate, fructose 6-phosphate, fructose 1,6-bisphosphate, dihydroxyacetone phosphate and 3-phospho-glycerate caused by overexpression of miR-500a-3p was found (Fig. 3E and Additional file 2: Fig. S3A–E). We then measured the expression of major genes involved in glucose transport and glycolysis. miR-500a-3p mimics reduced the RNA level of *HK2*, *PFKP*, *PGK1* and *LDHA* (Fig. 3F) and the protein level of GLUT1, HK2 and PKM2 (Fig. 3G). Since both RNA and protein levels of HK2 were downregulated by miR-500a-3p, HK2 was evaluated in the CRC cohort. It was negatively correlated with the miR-500a-3p levels (r = − 0.418, *P* < 0.0001, Fig. 3H, I), confirming the negative regulation of HK2 by miR-500a-3p in CRC tissues. These data suggest that the overexpression of miR-500a-3p causes a decrease in glucose metabolism via the regulation of multiple genes involved in glycolysis.

### CDK6 is a direct target and down regulated by miR-500a-3p

**Fig. 4:**
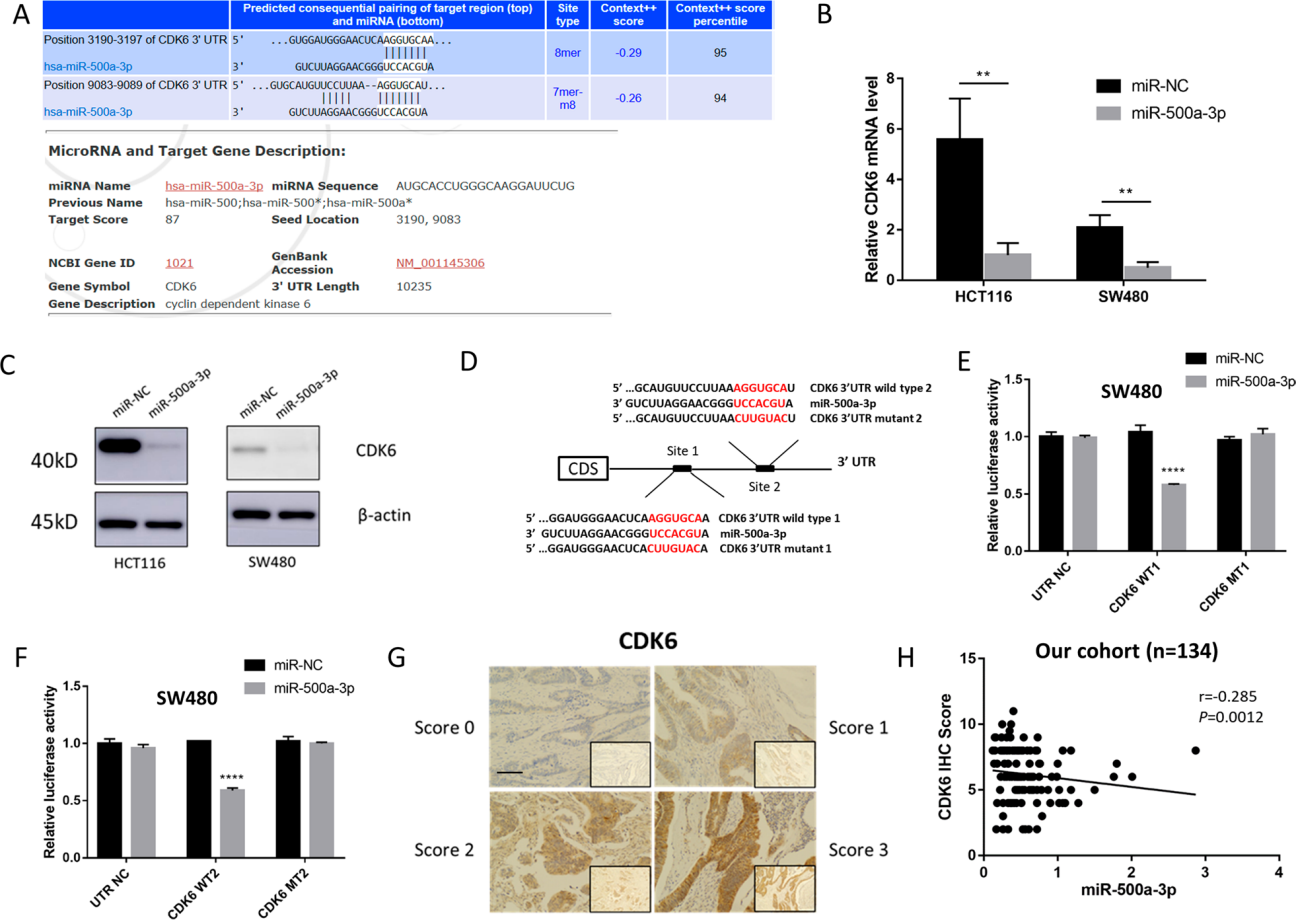
CDK6 is the direct target and down regulated by miR-500a-3p. **A** Potential binding sites predicted by Targetscan and miRDB between miR-500a-3p and CDK6 mRNA. **B** CDK6 mRNA levels after transfected with miR-500a-3p in HCT116 and SW480 cells by qPCR. The data were presented as the means ± SD (n = 3). **C** CDK6 protein levels after transfected with miR-500a-3p in HCT-116, SW480 and HT29 cells by western blot. **D** Schematic representation of luciferase reporter construct containing CDK6 3′ UTR with either wild type (WT) or mutant (MT) miR-500a-3p target site. Binding sites were marked in red. **E**, **F** The luciferase activity was detected in SW480 cells co-transfected with CDK6-WT or CDK6-MT and miR-NC or miR-500a-3p. The data were presented as the means ± SD (n = 3). **G** The specimens in the cohort which contained 134 continuous CRC patients were stained with CDK6. Scale bar, 100 μm. **H** Association between miR-500a-3p mRNA level and CDK6 IHC score were conducted in the cohort (n = 134) (Spearman’s correlation analysis). **P < 0.01, ****P < 0.0001

To explore the mechanisms by which miR-500a-3p inhibits the proliferation and glycolysis of CRC, we firstly used the publicly available algorithms TargetScan and miRDB to find the potential binding sites of glycolytic enzymes, like HK2, PFKP, PGK1 and LDHA, that are downregulated by miR-500a-3p. However, their 3′UTR did not have the conserved binding site of miR-500a-3p, indicating it may regulate glycolysis indirectly (Additional file 2: Fig. S4A). CDK6, a novel glycolysis regulator [17], was predicted as a target of miR-500a-3p by TargetScan and miRDB database (Fig. 4A). qPCR and Western blotting analysis showed that miR-500a-3p reduced both mRNA and the protein level of CDK6, indicating that CDK6 may act as a downstream target of miR-500a-3p (Fig. 4B, C). To further confirm this, we constructed two pairs of luciferase reporter plasmids containing the predicted seed sequence of miR-500a-3p in the 3′-UTR of the CDK6 mRNA and a control reporter containing the mutated sequence of the same fragment (Fig. 4D). miR-500a-3p significantly inhibited the luciferase activity in cells containing the wild-type CDK6 sequence, but not the mutant sequence (Fig. 4E, F). Finally, we evaluated the protein level of CDK6 in clinical CRC samples using IHC (Fig. 4G), and a significant negative correlation was revealed between miR-500a-3p and CDK6 expression (*P* = 0.0012, r = − 0.285, Fig. 4H). Collectively, these findings suggest that CDK6 is a direct target of miR-500a-3p in CRC.

### CDK6 is upregulated and promotes aerobic glycolysis in CRC

**Fig. 5:**
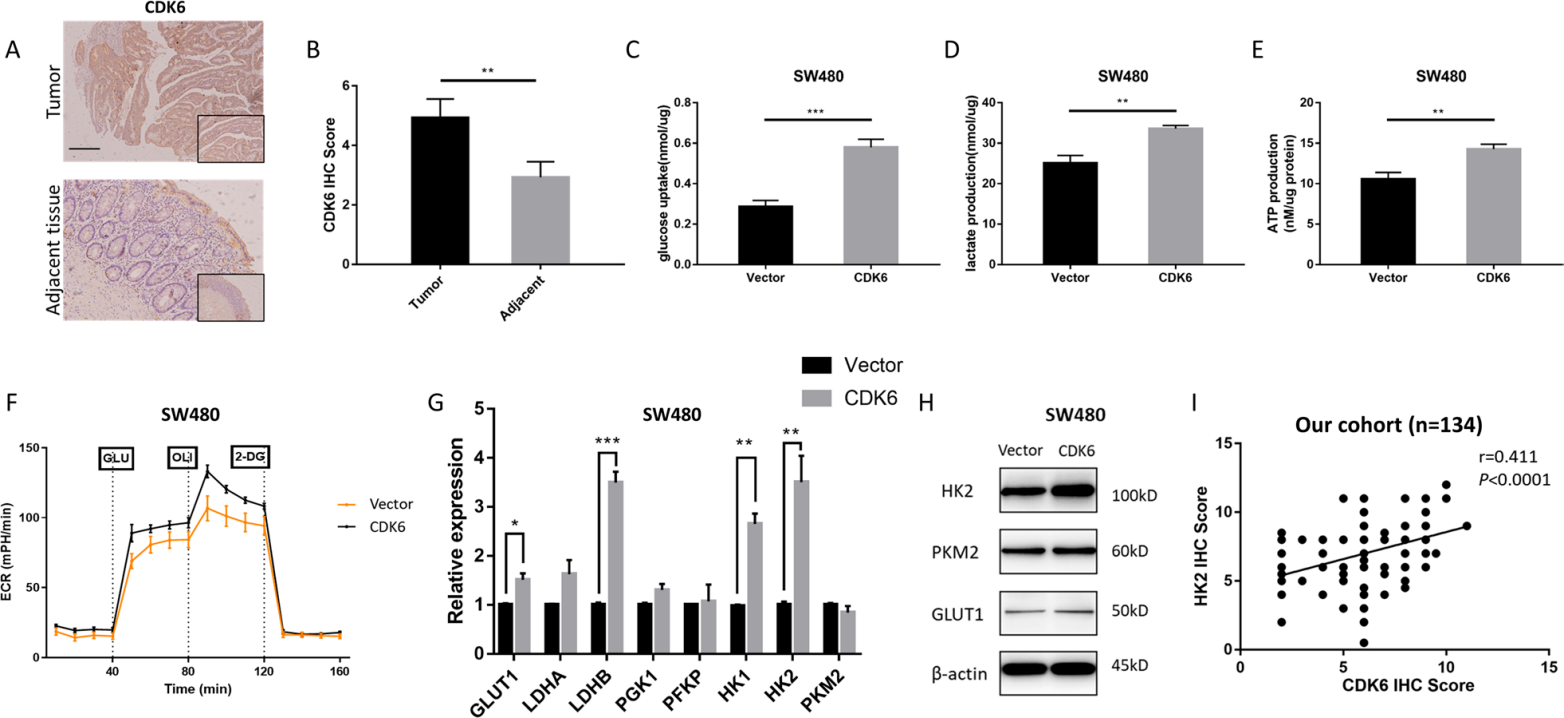
CDK6 is upregulated and promotes aerobic glycolysis in CRC. **A**, **B** The abundance of CDK6 was examined in CRC tissue samples (n = 20) and adjacent normal tissue samples (n = 20) by IHC staining. Scale bar, 100 μm. Representative images (left) and quantification (right) were shown. **C**–**E** The glucose uptake (**C**), lactate production (**D**), intracellular ATP (**E**) were measured in SW480 cells after transfected with vector and CDK6. **F** The extracellular acidification rate (ECAR) of SW480 cells were analyzed by Seahorse XF96 Extracellular Flux Analyzer after transfected with vector and CDK6. Data were shown as the mean ± SD of three independent experiments. **G**, **H** The mRNA level of GLUT1, LDHB, HK1 and HK2 (**G**) by qPCR and the protein level of GLUT1 and HK2 (**H**) by western blot in SW480 cells transfected with vector and CDK6. **I** Spearman correlation between IHC score of CDK6 and HK2 were conducted in the cohort (n = 134). *P < 0.05, **P < 0.01, ***P < 0.001

We examined CDK6 protein levels in 20 paired human CRC tumors and adjacent nontumor tissues using IHC (Fig. 5A). The IHC staining results revealed that it was significantly upregulated in CRC tissues compared to nontumor tissues (Fig. 5B). Survival analysis of a large CRC cohort showed that high CDK6 expression was significantly correlated with poor overall survival (*P* = 0.0333, Additional file 2: Fig. S5A) and disease-free survival (*P* = 0.0559, Additional file 2: Fig. S5B). CCK-8 and cell cycle assays indicated that CDK6 enhanced CRC cell proliferation (Additional file 2: Fig. S5C, D). Then, we explored the influence of CDK6 on the aerobic glycolysis of CRC cells. As exhibited in Fig. 5C–F, CDK6 promoted glucose uptake, the production of lactate and ATP and the ECAR in SW480 cells. The expression of major genes involved in glucose transport and glycolysis were also measured. The RNA level of *GLUT1*, *LDHB*, *HK1* and *HK2* and the protein level of GLUT1, HK2 were elevated after overexpressing CDK6 (Fig. 5G, H). In CRC tissues, the IHC scores of HK2 was positively correlated with that of CDK6 (r = 0.411, *P* < 0.0001, Fig. 5I). These findings indicated that CDK6 plays an important role in promoting aerobic glycolysis in CRC.

### miR-500a-3p mediates the inhibition of proliferation and aerobic glycolysis in CRC cells by targeting CDK6

**Fig. 6:**
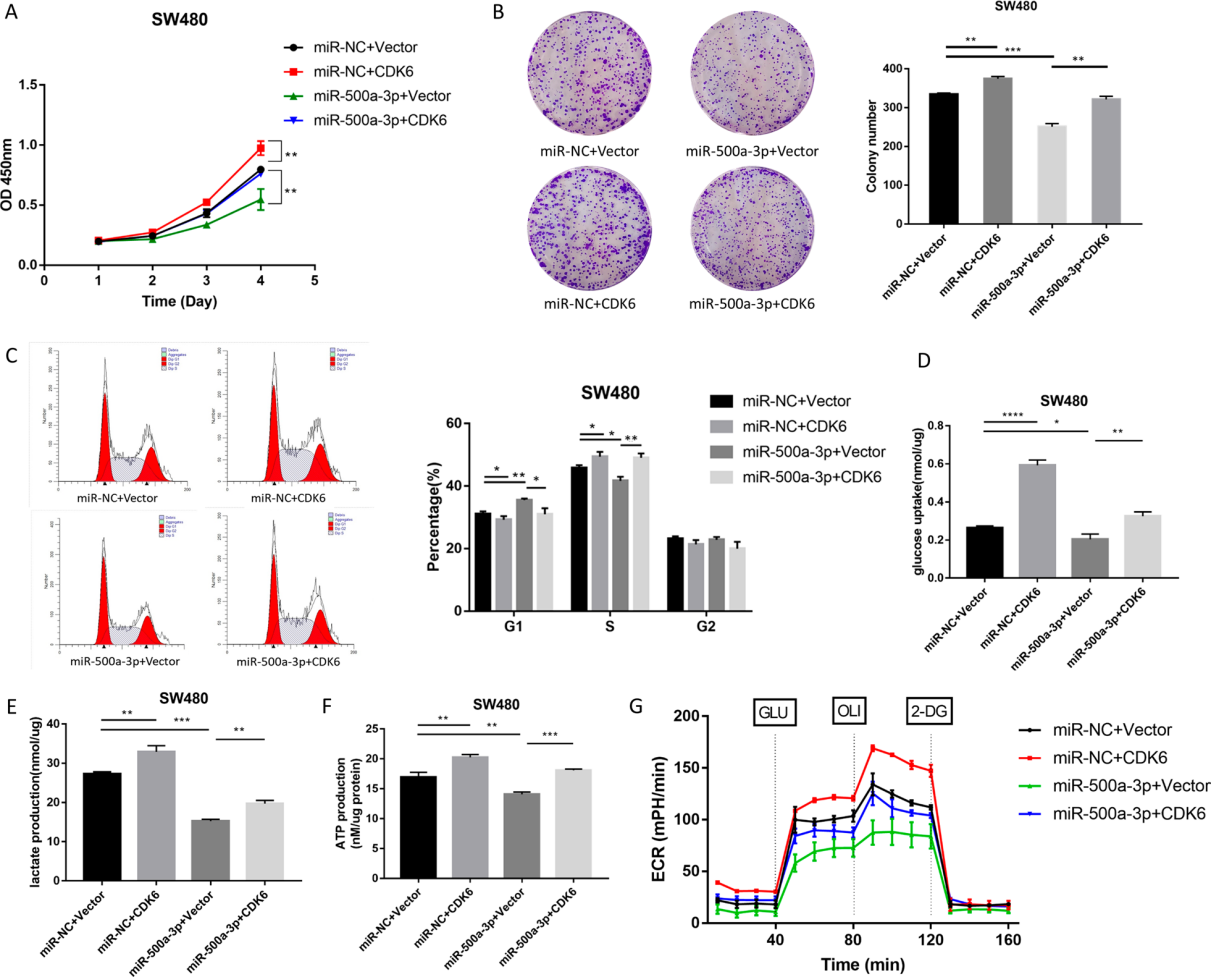
CDK6 overexpression recovers the proliferation and glycolysis of CRC cells which is restrained by the transfection of miR-500a-3p mimics. **A**–**G** SW480 cells were transfected with miR-NC, miR-500a-3p, miR-500a-3p + vector or miR-500a-3p + CDK6. **A** The proliferation ability of SW480 cells after transfection was measured by CCk-8 assay. **B** The colony formation ability of after transfection were measured by colony formation assay. Representative images (left) and quantification (right) were shown. **C** The cell cycle was evaluated by flow cytometry after transfection. Representative images (left) and quantification data (right) were shown. **D**–**F** The glucose uptake (**D**), lactate production (**E**), intracellular ATP (**F**) in SW480 cells were measured after transfection using Glucose Uptake Colorimetric Assay kit, Lactate Assay Kit and ATP Colorimetric Assay kit. **G** Seahorse XF 96 Extracellular Flux Analyzer was used to detect the ECAR in four groups of SW480 cells. Data were shown as the mean ± SD of three independent experiments. *P < 0.05, **P < 0.01, ***P < 0.001, ****P < 0.0001

To address whether miR-500a-3p exerted its role through targeting CDK6, we conducted rescue experiments. The CCK-8 and colony formation assays showed that CDK6 promoted CRC cell proliferation and colony formation and offset the inhibitory effect caused by miR-500a-3p (Fig. 6A, B). As shown in Fig. 6C, CDK6 overexpression counteracted the inhibitory effect of miR-500a-3p on the cell cycle of CRC cells. We explored the influence of miR-500a-3p/CDK6 axis on the aerobic glycolysis of CRC cells by measuring glucose uptake, lactate production, intracellular ATP level and ECAR. The inhibition of glycolysis caused by miR-500a-3p could be partly eliminated by CDK6 overexpression (Fig. 6D–G). Together, these results indicated that miR-500a-3p suppressed CRC proliferation and aerobic glycolysis through down-regulating CDK6.

## Materials and methods

### Patients

From January 2015 to March 2015, 134 continuous CRC specimens and 20 matching adjacent non-tumor specimens obtained from CRC patients at the Zhongshan Hospital, Fudan University were collected. The inclusion criteria were as follows: with histologically confirmed CRC, receiving radical surgery, having complete follow-up. The exclusion criteria were receiving preoperative chemotherapy or radiation, pregnancy or breastfeeding, previous malignancy within 5 years or no written informed consent. The primary tumor ki67 score is the routine pathological evaluation of our center and was evaluated by two experienced pathologists. Patients in the cohort (134 continuous CRC specimens) who received PETCT preoperatively (n = 52) were included to analyze the association between miR-500a-3p expression and the SUV of the primary tumors. The study was authorized by the Ethics Committee of the Zhongshan Hospital, Fudan University. Written informed consent was obtained from each patient.

### Collection of TCGA public data and identification of significantly differential expression miRNA

We collected gene expression profiles of 551 COADREAD samples from the TCGA cohort (http://gdac.broadinstitute.org/). Data including mRNA expression level and miRNA expression level. All the data have been standardized. For miRNAs differential analysis, we calculated the mean expression level of these miRNAs and divided them into high and low expression groups.

The R package DESeq2 (version 1.22.2) was used to calculate the differential expressed t statistics for microarray and RNA sequencing data. We used the univariate Cox proportional hazards model to examine the associations between gene expression and overall survival. MiRNAs with P values less than 0.05 were considered to be statistically significant and included in consensus survival analysis.

### Immunohistochemistry staining

The whole cohort which contained 134 continuous CRC specimens were stained with the CDK6 antibody (ab124821, Abcam) and HK2 antibody (2867T, Cell Signaling Technology). The 20 matched adjacent non-tumor specimens were stained with CDK6 antibody (ab124821, Abcam). IHC scores were conducted according to the ratio and intensity of positive-staining areas. The staining areas were scored as follow: 0–25%, score 1; 25–50%, score 2; 50–75%, score 3; and beyond 75%, score 4. The signal intensity was scored on a scale of 0–3: 0-negative, 1-weak, 2-moderate and 3-strong. IHC scores were averaged from two experienced pathologists who scored the IHC staining independently.

### Cell lines and culture

Human CRC cell lines [HCT116 (RRID: CVCL_0291) and SW480 (RRID: CVCL_0546)] and normal human colon epithelial cell line NCM460 (RRID: CVCL_0460) were obtained from the National Collection of Authenticated Cell Cultures (Shanghai, China). All human cell lines have been authenticated using STR profiling within the last three years and all experiments were performed with mycoplasma-free cells. These cell lines were maintained in Dulbecco’s Modified Eagle Medium (Logan Utah, HyClone, USA) with 10% fetal bovine serum (FBS; Gibco), 1% penicillin (10 U/mL) and 1% streptomycin (10 μg/mL) in an incubator with the environment of 37 °C and 5% CO_2_.

### Quantitative real-time polymerase chain reaction (qRT-PCR)

RNA was isolated with Trizol reagent. The purity and the concentration of different RNA samples were measured using NanoDrop ND-1000 (Thermo Scientific, USA). Reverse transcription reaction was performed using miRcute miRNA cDNA synthesis Kit (kr211, Tiangen, China) for miRNAs and SuperScript IV VILO cDNA synthesis kit (11756050, Thermo Scientific, USA) for mRNAs. Quantitative RT-PCR (qRT-PCR) was performed using SYBR® Premix Ex Taq™ (Takara) containing mRQ 3′ Primer on an ABI 7500 platform (Applied Biosystems, Carlsbad, CA, USA). The relative quantities of miR-500a-3p in cells were normalized to U6. Beta-actin was used as an internal control for mRNA detection. The primer sequences for miRNA and mRNA detection are listed in Additional file 1: Table S3.

### Lentivirus and miRNA transfection

The lentivirus particles overexpressing human miR-500a-3p and CDK6 were purchased from Genomeditech (Shanghai, China). miR-500a-3p mimics were synthesized by Genomeditech (Shanghai, China). The sequences of miR-500a-3p mimic were 5′-AUGCACCUGGGCAAGGAUUCUG-3′. The miRNA oligonucleotides were transfected using Lipofectamine® RNAiMAX™ (50 nmol/L; Invitrogen, CA, USA).

### Cell viability assay

At 72 h after lentivirus infection or at 24 h after miRNA transfection, HCT-116 (2500/well) and SW480 (2000/well) cells were plated into 96-well culture plates. Cell viability was measured at 24, 48, 72 and 96 h post-seeding, using the Cell Counting Kit-8 (Dojindo, Kumamoto, Japan) according to the manufacturer’s instructions.

### Colony-formation assay

HCT-116 (1500/well) and SW480 (2000/well) cells were seeded in six-well plates after 72 h lentivirus infection. The culture medium was changed every 3 days. After incubating for 2 weeks, colonies were fixed with 4% paraformaldehyde for 15 min and stained with 5% Giemsa for 20 min. The colonies containing at least 50 cells were scored.

### Cell cycle analysis

Seventy-two hours after infection, cells were harvested, washed with phosphate-buffered saline (PBS), and fixed in 70% ethanol at 4 °C overnight. After fixation, cells were washed with PBS before suspension in RNase A/propidium iodide solutions (100 mg/mL RNase A and 5 μg/mL propidium iodide). Cells were incubated at room temperature for an hour. Stained cells were analyzed by a FACScan flow cytometer (BD Biosciences, Mountain View, CA, USA).

### Cell apoptosis analysis

Cell apoptosis was assessed by annexin V/propidium iodide (BD Biosciences, San Jose, CA, USA). Cells were harvested, washed with PBS, and resuspended in 1× binding buffer at a concentration of 1 × 10^6^ cells/mL. A total of 100 μL solution was transferred to a new tube and added with 5 μL of APC Annexin V and 5 μL of propidium iodide. Cells were incubated at room temperature for 15 min in the dark and then analyzed by a FACScan flow cytometer.

### Cell invasion assay

The cell invasion assay was performed in 24-well transwell chambers pre-coated with Matrigel (Corning, NY, USA). HCT-116 cells (10^5^/well). Serum-free medium (200 μL) was placed into the upper chamber, and 600 μL complete medium (with 10% FBS) was filled into the lower chamber. Cells on the inner membrane were removed with a cotton swab after 24 h. The outer membrane was fixed in 4% formaldehyde (in PBS) and stained with 0.5% crystal violet. Cell numbers were counted and averaged in five random fields at a magnification of 100×.

### Xenograft tumor assay

BALB/c nude mice (6 weeks old) were purchased from Shanghai SLAC Laboratory animal co. ltd (shanghai, China) and randomly divided into 2 groups (n = 7/6). SW480 (3 × 10^6^) or HCT116 (4 × 10^6^) cells that stably transfected with miR-NC or miR-500a-3p were injected at the back region of BALB/c mice subcutaneously. The volume of tumors was measured twice a week using a vernier caliper with the method of volume = (length × width^2^)/2. The mice were killed after inoculation for 30 d, and the tumors were weighed. Tumor tissues were subjected to the expression analysis of ki67. The procedures in this study were permitted by the Animal Research Committee of the Zhongshan Hospital, Fudan University.

### Luciferase reporter assay

The putative miR-500a-3p binding sequence and the matching mutant binding sequence in the 3′untranslated region (3′UTR) of CDK6 were amplified and inserted into pGL3 luciferase reporter vector (Genomeditech, shanghai, China). SW480 cells were transfected with miR-NC or miR-500a-3p and the above-constructed reporter plasmids. After transfection for 48 h, the luciferase activities in different groups were determined by the dual-luciferase reporter assay system (Promega).

### Glucose, lactate and ATP measurement

SW480 and HCT116 cells were cultured with an FBS-free medium and culture medium was collected after 24 h. Glucose Uptake Colorimetric Assay kit (Biovision, Milpitas, California, USA), Lactate Assay Kit (Biovision) and ATP Colorimetric Assay kit (Biovision) were utilized to detect the glucose consumption, the lactate production and ATP in CRC cells according to the manufacturer’s instructions. The glucose, lactate and ATP levels were normalized to total cell protein.

### Seahorse analysis

Extracellular acidification rate (ECAR) was detected using a Seahorse XF96 analyzer (Seahorse Biosciences, USA). SW480 cells (10^5^/well) were seeded in a 96-well XF96 microplate (Seahorse Biosciences, USA). Before experiments, cell culture medium was replaced and cells were then incubated with assay medium for 1 h at 37 °C in a CO_2_-free incubator. ECAR was detected using a sequential injection of 10 mM glucose, 2 mM oligomycin (Sigma-Aldrich) and 50 mM 2-deoxyglucose (2-DG, Sigma-Aldrich). Each cycle of measurement involved mixing (3 min), waiting (2 min), and measuring (3 min) cycles.

### Mass spectrometry

The targeted metabolomics analyses were performed using an HPLC system (Agilent 1290, Agilent Technologies) and a mass spectrometer (Agilent 5500, Agilent Technologies). A 10-cm dish of cultured tumor cells was collected, adding 1 mL acetonitrile/methanol water (v, 2:2:1) and storing at − 80℃ after quick freezing in liquid nitrogen. Sample preparation processes were performed in accordance with the above method of parallel preparation of QC samples. MRM transitions representing the metabolites were simultaneously monitored, and the positive/negative polarity switching was used. Data analyses were performed as instructed by Shanghai Applied Protein Technology [18].

### Western-blot

Whole protein extracts were lysed by radioimmunoprecipitation assay (RIPA) buffer (Thermo Fisher Scientific) according to the manufacturer’s protocol. At that time, 30 μg proteins were run on a 10% sodium dodecyl sulfate polyacrylamide gel electrophoresis (SDS-PAGE) gel at 100 V for 2 h and transferred to a polyvinylidene fluoride membrane at 80 V for 2 h. Membranes were blocked with 5% bovine serum albumin (BSA) in Tris-buffered saline with Tween-20 (TBST) at room temperature for 1 h. They were then incubated overnight at 4 °C with one of the following primary antibodies: anti-CDK6 (ab124821, Abcam) (1:1000), anti-GLUT1 (ab115730, Abcam) (1:1000) and anti-HK2 (2867T, Cell Signaling Technology) (1:1000) and anti-beta actin (1:1000) from Santa Cruz Biotechnology (Dallas, Texas, USA). After washing with TBST, membranes were incubated with secondary antibodies for 1 h at room temperature and signals were developed using an enhanced chemiluminescence kit (Pierce, Waltham, MA, USA).

### Statistical analysis

SPSS (version 22.0, SPSS Inc.) or GraphPad Prism software (version 7.0, USA) were used to analyze the data and generate the graphs. The differences between the miR-500a-3p expression and the clinical characteristics of CRC patients were analyzed using χ^2^ test. Survival curves were generated using the Kaplan–Meier method and log-rank tests. Univariate and multivariate Cox regression analyses were conducted to identify the independent factors. The correlation among the expression of miR-500a-3p, CDK6 and HK2 was analyzed using Spearman’s correlation test. Student’s t-test or the Mann–Whitney U test was used to calculate the *P* values between two groups. A value of *P* < 0.05 was identified as statistically significant. R software 3.5.1 was applied to this study for the statistical analyses. All miRNA expression data were normalized.

## Discussion

Both investigating new prognostic biomarkers and uncovering the mechanisms behind the progression of CRC are critical for developing CRC therapies. In the current study, we focused on the role of miR-500a-3p in CRC progression. We found that patients with low miR-500a-3p expression had a worse prognosis and miR-500a-3p may represent an independent prognostic factor for CRC patients. Further in vitro and in vivo experiments showed that miR-500a-3p inhibits CRC cell proliferation and aerobic glycolysis. In addition, we discovered CDK6 as a novel downstream target of miR-500a-3p. These data suggested that miR-500a-3p might serve as a novel prognostic biomarker or therapeutic target of CRC.

Accumulating evidence indicates that miRNAs contribute to CRC progression [19–21]. However, research studies screening miRNAs from the sequencing results of large cohorts were relatively rare. We analyzed miRNAs that are significantly related to the prognosis of CRC from the cBioportal database and found that miR-500 has the most significant correlation with the prognosis of CRC patients. Then we focused on miR-500a-3p which has not been reported in CRC.

Interestingly, both tumor-promoting and—suppressive roles of miR-500a-3p have been revealed. In both hepatocellular and gastric carcinoma, miR-500a-3p served as a tumor promoter via regulating the cancer cell stemness [11, 12]. While in non-small cell lung cancer, miR-500a-3p acts as a tumor suppressor via downregulating LY6K expression [22]. The distinct roles of miR-500a-3p might be due to the heterogeneity of tumors and differences of cancer types. In this study, we provide new evidence that miR-500a-3p serves as a tumor suppressor in CRC and more importantly, for the first time, reveal the role of miR-500a-3p on cancer cell metabolism. Specifically, we found that miR-500a-3p downregulated the expression of glycolytic enzymes and inhibited glycolysis. Decreased glycolysis restrains the cellular buildings for rapid cell proliferation [4], and ultimately contributes to the tumor-suppressive role of miR-500a-3p in CRC.

Previous studies have shown that CDK6 is regulated by several miRNAs, such as miR-29b, miR-211 and miR-497 [23–25]. Our study provided additional evidence to support that CDK6 is regulated by miRNAs. We report that CDK6 is a direct functional target of miR-500a-3p in CRC and found that miR-500a-3p inhibits the transcription of CDK6. In different cancer types, CDK6 may regulate different metabolic enzymes and play distinct roles in glycolysis. Wang et al. found that CDK6 inhibits the glycolytic pathway and re-directs the glycolytic intermediates into the pentose phosphate pathway (PPP) and serine pathways [17]. Xing et al. proved that CDK6 promotes glycolysis via phosphorylation of the fructose bisphosphate PFK2 (PFKFB3) in breast cancer [26]. In this study, we found that CDK6 enhanced glycolysis in CRC and HK2 might be a potential downstream target of CDK6.

The inhibition of glycolysis by miR-500a-3p was only partially restored by CDK6 overexpression, indicating other targets might also be involved in miR-500a-3p-mediated regulation of glycolysis. Some reported targets of miR-500a-3p, such as XBP1 and FBXW7, may regulate glycolysis directly or indirectly [11, 27–29]. Further studies may be needed to validate the mechanistic link of miR-500a-3p and these targets in modulating glycolysis in CRC.

## Conclusion

Taken together, miR-500a-3p was found to be a tumor suppressor in CRC. miR-500a-3p suppressed the proliferation, cell cycle and glycolysis of CRC cells partially via CDK6. These findings will expand our understanding of the versatile role of miR-500a-3p in CRC progression and support the rationale of miR-500a-3p in CRC treatment.

## Supplementary Information

**Additional file 1: Table S1.** MiRNAs that significantly affect the prognosis of CRC patients in cBioportal database. **Table S2.** Univariate and multivariate analysis showed miR-500a-3p expression was an independent prognostic factor in CRC paients. **Table S3.** Primer sequences for miRNA and mRNA detection.

**Additional file 2: Figure S1.** (A, B) Low miR-500 expression was significantly associated with poor overall survival (A) and progression-free survival (B) of CRC patients in cBioportal database. **Figure S2.** (A) qRT-PCR was carried out to detect the level of miR-500a-3p in SW480 and HCT116 cells transfected with miR-NC or miR-500a-3p. (B) miR-500a-3p mimics did not increase apoptosis in HCT116 cells. (C) miR-500a-3p mimics inhibited cell migration in HCT116 cells. (D, E) Verification of miR-500a-3p overexpression efficiency in SW480 and HCT116 cells. (F) Representative tumor diagrams in different groups were shown. (G, H) Tumor volume and weight in the miR-500a-3p group were significantly lower than those in the miR-NC group. (I) Ki-67 expression was significantly higher in tumors of miR-500a-3p group than that of miR-NC group. **Figure S3.** (A–E) Glucose 6-phosphate (A), fructose 6-phosphate (B), fructose 1,6-bisphosphate (C), dihydroxyacetone phosphate (D) and 3-phospho-glycerate (E) in the miR-500a-3p group were significantly lower than those in the miR-NC group. **Figure S4.** The 3′UTR of glycolysis enzymes did not have conserved binding site of miR-500a-3p. **Figure S5.** (A, B) CDK6 high expression was significantly associated with poor overall survival (A) and disease-free survival (B) in CRC specimens. (C, D) CDK6 enhanced CRC cell proliferation using CCK-8 (C) and cell cycle assays (D). **Figure S6.** (A) The correlation between miR-500a-3p and CDK6 in CPTAC_COAD database (n = 105). (B) The correlation between miR-500a-3p and HK2 in CPTAC_COAD database (n = 105). (C) The representative images of RNA electrophoresis.

## Data Availability

All data generated or analysed during this study are included in this published article and its Additional files.

## Abbreviations

CRC: Colorectal cancer
qPCR: Quantitative real-time PCR
IHC: Immunohistochemistry
CCK-8: Cell counting kit 8
miRNAs: MicroRNAs
CDK6: Cyclin-dependent kinases 6
SUV: Standard Uptake Value
FACS: Fluorescence-activated cell sorting
ECAR: Extracellular acidification rate
PPP: Pentose phosphate pathway
HK2: Hexokinase2
YY1: Yin Yang 1
HDAC2: Histone deacetylase 2
CI: Confidence interval
ATP: Adenosine-triphosphate
PFKP: Platelet isoform of phosphofructokinase
PGK1: Phosphoglycerate kinase 1
LDHA: Lactate dehydrogenase-A
GLUT1: Glucose transporter 1
PKM2: Pyruvate kinase M2
3′UTR: 3′Untranslated region
LDHB: Lactate dehydrogenase-B
PETCT: Positron emission tomography computed tomography
TCGA: The Cancer Genome Atlas
FBS: Fetal bovine serum
PBS: Phosphate-buffered saline
2-DG: 2-Deoxyglucose
RIPA: Radioimmunoprecipitation assay
SDS-PAGE: Sodium dodecyl sulfate poly-acrylamide gel electrophoresis
BSA: Bovine serum albumin
TBST: Tris buffered saline with Tween-20
LY6K: Lymphocyte antigen 6 complex locus K
PFKFB3: 6-Phosphofructo-2-kinase/fructose-2,6-biphosphatase 3
XBP1: X-box binding protein 1
FBXW7: F-box and WD repeat domain containing 7
