# Supplementary material for: Activation of miR-500a-3p/CDK6 axis suppresses aerobic glycolysis and colorectal cancer progression
Source: J Transl Med. 2022 Mar 3;20:106. doi: 10.1186/s12967-022-03308-8 (PMC8896266; doi:10.1186/s12967-022-03308-8)
Supplement: Supplementary file 1 — Additional file 1: Table S1. MiRNAs that significantly affect the prognosis of CRC patients in cBioportal database. Table S2. Univariate and multivariate analysis showed miR-500a-3p expression was an independent prognostic factor in CRC paients. Table S3. Primer sequences for miRNA and mRNA detection. [file 12967_2022_3308_MOESM1_ESM.docx]

Table S1: MiRNAs that significantly affect the prognosis of CRC patients in cBioportal database

|  | *P* value for OS | *P* value for PFS |
| --- | --- | --- |
| **hsa-mir-500a** | 0.007962 | 0.000159 |
| **hsa-mir-378** | 0.011046 | 0.000321 |
| **hsa-mir-942** | 0.013058 | 0.047164 |
| **hsa-mir-25** | 0.024919 | 0.005457 |
| **hsa-mir-93** | 0.026128 | 0.011809 |
| **hsa-mir-501** | 0.04222 | 0.017286 |
| **hsa-mir-320d-2** | 0.048052 | 0.03071 |
| **hsa-mir-215** | 0.207932 | 0.034339 |
| **hsa-mir-7-3** | 0.239218 | 0.028213 |
| **hsa-mir-218-1** | 0.272762 | 0.013947 |
| **hsa-mir-660** | 0.2797 | 0.030309 |
| **hsa-mir-629** | 0.362169 | 0.003125 |
| **hsa-mir-362** | 0.377279 | 0.037212 |
| **hsa-mir-133b** | 0.393009 | 0.034436 |
| **hsa-mir-130b** | 0.441445 | 0.01633 |
| **hsa-mir-1197** | 0.501451 | 0.029192 |
| **hsa-mir-137** | 0.534776 | 0.020828 |
| **hsa-mir-224** | 0.570264 | 0.047566 |
| **hsa-mir-502** | 0.638827 | 0.005797 |
| **hsa-mir-194-1** | 0.651453 | 0.009502 |
| **hsa-mir-548o** | 0.666111 | 0.01829 |
| **hsa-mir-194-2** | 0.700261 | 0.002816 |
| **hsa-mir-16-1** | 0.77616 | 0.016482 |
| **hsa-mir-1306** | 0.779533 | 0.04002 |
| **hsa-mir-550-1** | 0.78285 | 0.011193 |
| **hsa-mir-221** | 0.847461 | 0.045595 |
| **hsa-mir-30e** | 0.848499 | 0.019438 |
| **hsa-mir-1307** | 0.868854 | 0.014851 |
| **hsa-mir-455** | 0.871657 | 0.024271 |
| **hsa-mir-532** | 0.876667 | 0.015801 |
| **hsa-mir-1977** | 0.880873 | 0.037482 |
| **hsa-mir-147b** | 0.888161 | 0.043134 |
| **hsa-mir-192** | 0.913342 | 0.005382 |
| **hsa-mir-628** | 0.914964 | 0.020949 |
| **hsa-mir-576** | 0.921696 | 0.004501 |
| **hsa-mir-372** | 0.944583 | 0.019322 |
| **hsa-mir-186** | 0.963606 | 0.007774 |
| **hsa-let-7f-1** | 0.976194 | 0.003495 |

Table S2: Univariate and multivariate analysis showed miR-500a-3p expression was an independent prognostic factor in CRC paients

|  | Univariate analysis | | | Multivariate analysis | |
| --- | --- | --- | --- | --- | --- |
|  | Hazard ratio, 95%CI | P | | Hazard ratio, 95%CI | P |
| **Age** |  | 0.198 |  | |  |
| **>65** | 2.316 (0.639-8.727) |  |  | |  |
| **≤65** | Ref |  |  | |  |
| **Gender** |  | 0.191 |  | |  |
| **Male** | 2.754 (0.603-12.576) |  |  | |  |
| **Female** | Ref |  |  | |  |
| **Location** |  | 0.260 |  | |  |
| **Left** | 0.034 (0.000-12.228) |  |  | |  |
| **Right** | Ref |  |  | |  |
| **Tumor size** |  | 0.227 |  | | 0.099 |
| **>5cm** | 2.238 (0.606-8.268) |  | 3.368 (0.796-14.252) | |  |
| **≤5cm** | Ref |  | Ref | |  |
| **Differentiation** |  | 0.808 |  | | 0.647 |
| **Poor** | 1.176 (0.318-4.350) |  | 0.720 (0.177-2.936) | |  |
| **High/moderate** | Ref |  | Ref | |  |
| **T stage** |  | 0.002 |  | | 0.032 |
| **T3-4** | 5.465 (1.875-15.928) |  | 4.532 (1.137-18.060) | |  |
| **T1-2** | Ref |  | Ref | |  |
| **N stage** |  | 0.000 |  | | 0.066 |
| **N1-2** | 4.550 (2.022-10.238) |  | 2.244 (0.949-5.304) | |  |
| **N0** | Ref |  | Ref | |  |
| **M stage** |  | 0.005 |  | | 0.012 |
| **M1** | 4.978 (1.605-15.443) |  | 6.076 (1.494-24.721) | |  |
| **M0** | Ref |  | Ref | |  |
| **Ras status** |  | 0.552 |  | |  |
| **Mutant** | 1.493 (0.399-5.587) |  |  | |  |
| **Wide type** | Ref |  |  | |  |
| **MiR-500a-3p expression** |  | 0.018 |  | | 0.037 |
| **High** | 0.084 (0.011-0.655) |  | 0.109 (0.014-0.875) | |  |
| **Low** | Ref |  | Ref | |  |

Table S3: Primer sequences for miRNA and mRNA detection

| hsa-miR-500a-3p-F^*^ | 5'-ATGCACCTGGGCAAGGATTCTG-3' |
| --- | --- |
| U6-F | 5'-CGCTTCGGCAGCACATATAC-3' |
| U6-R | 5'-CGAATTTGCGTGTCATCCTT-3' |
| β-Actin-R | 5'-GCCGTGGTGGTGAAGCTGT-3' |
| β-Actin-F | 5'-ACCCACACTGTGCCCATCTA-3' |
| LDHA-F | 5'-GACCTACGTGGCTTGGAAGAT-3' |
| LDHA-R | 5'-AGGACCCACCCATGACAGC-3' |
| LDHB-F | 5'-GCGGAAGAAGAGGCAACAGT-3' |
| LDHB-R | 5'-AGCAAGTTCATCAGCCAGAGAC-3' |
| PGK1-F | 5'-ATCTGCCACAGAAGGCTGGT-3' |
| PGK1-R | 5'-GAGCTGGATCTTGTCTGCAACT-3' |
| PFKP-F | 5'-GTGGACGGAGGCTCAAACAT-3' |
| PFKP-R | 5'-GGTTGCAAGCAGCCTTCAG-3' |
| HK1-F | 5'-AGTTGGATTGCAAGGACAGTGT-3' |
| HK1-R | 5'-AGAAGTGAGGCCGTCACAGC-3' |
| HK2-F | 5'-CGCATCTGCTTGCCTACTTC-3' |
| HK2-R | 5'-TTGCGGAACCGCTTAGAGA-3' |
| PKM2-F | 5'-TGACACATTCCTGGAGCACAT-3' |
| PKM2-R | 5'-GAAGTTCAGACGAGCCACATTC-3' |
| CDK6-F | 5'-GCTGACCAGCAGTACGAATG-3' |
| CDK6-R | 5'-GCACACATCAAACAACCTGACC-3' |

*: Reverse primer is included in the SYBR® Premix Ex Taq™ (Takara).
