# Supplementary material for: Activation of miR-500a-3p/CDK6 axis suppresses aerobic glycolysis and colorectal cancer progression
Source: J Transl Med. 2022 Mar 3;20:106. doi: 10.1186/s12967-022-03308-8 (PMC8896266; doi:10.1186/s12967-022-03308-8)
Supplement: Supplementary file 2 — Additional file 2: Figure S1. (A, B) Low miR-500 expression was significantly associated with poor overall survival (A) and progression-free survival (B) of CRC patients in cBioportal database. Figure S2. (A) qRT-PCR was carried out to detect the level of miR-500a-3p in SW480 and HCT116 cells transfected with miR-NC or miR-500a-3p. (B) miR-500a-3p mimics did not increase apoptosis in HCT116 cells. (C) miR-500a-3p mimics inhibited cell migration in HCT116 cells. (D, E) Verification of miR-500a-3p overexpression efficiency in SW480 and HCT116 cells. (F) Representative tumor diagrams in different groups were shown. (G, H) Tumor volume and weight in the miR-500a-3p group were significantly lower than those in the miR-NC group. (I) Ki-67 expression was significantly higher in tumors of miR-500a-3p group than that of miR-NC group. Figure S3. (A–E) Glucose 6-phosphate (A), fructose 6-phosphate (B), fructose 1,6-bisphosphate (C), dihydroxyacetone phosphate (D) and 3-phospho-glycerate (E) in the miR-500a-3p group were significantly lower than those in the miR-NC group. Figure S4. The 3′UTR of glycolysis enzymes did not have conserved binding site of miR-500a-3p. Figure S5. (A, B) CDK6 high expression was significantly associated with poor overall survival (A) and disease-free survival (B) in CRC specimens. (C, D) CDK6 enhanced CRC cell proliferation using CCK-8 (C) and cell cycle assays (D). Figure S6. (A) The correlation between miR-500a-3p and CDK6 in CPTAC_COAD database (n = 105). (B) The correlation between miR-500a-3p and HK2 in CPTAC_COAD database (n = 105). (C) The representative images of RNA electrophoresis. [file 12967_2022_3308_MOESM2_ESM.docx]

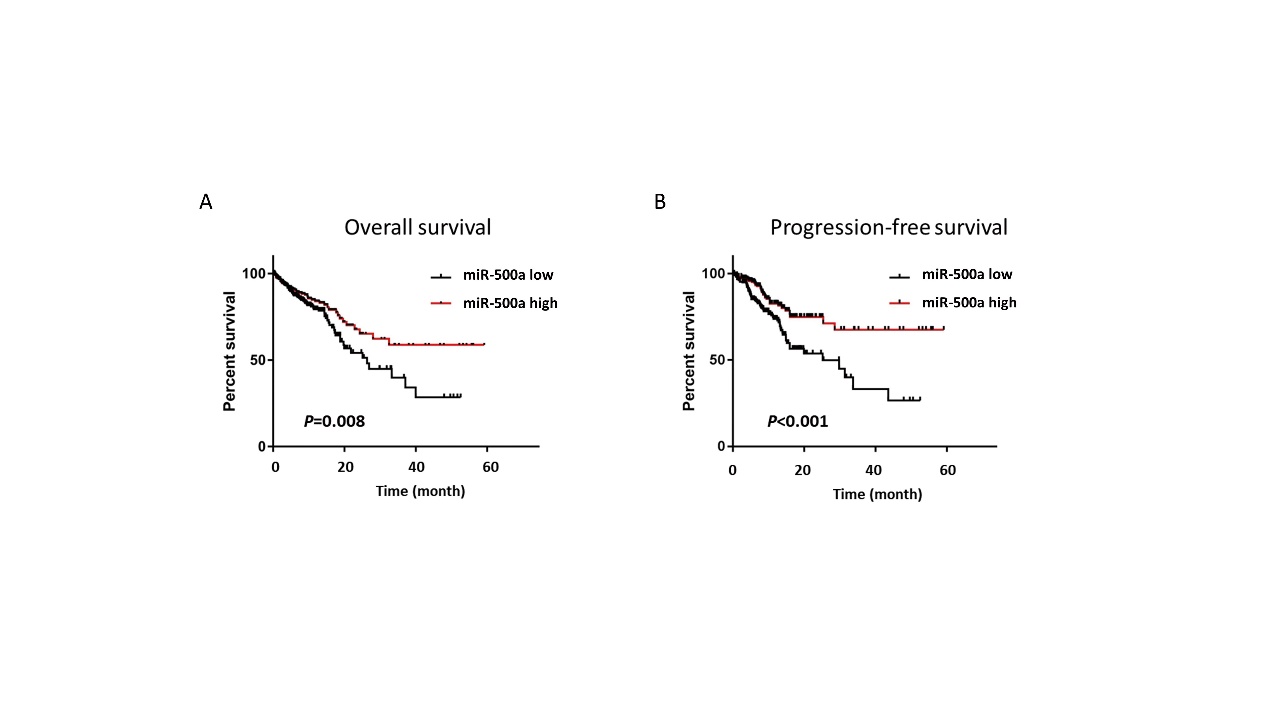
**Figure S1**

(A-B) Low miR-500 expression was significantly associated with poor overall survival (A) and progression-free survival (B) of CRC patients in cBioportal database.

**
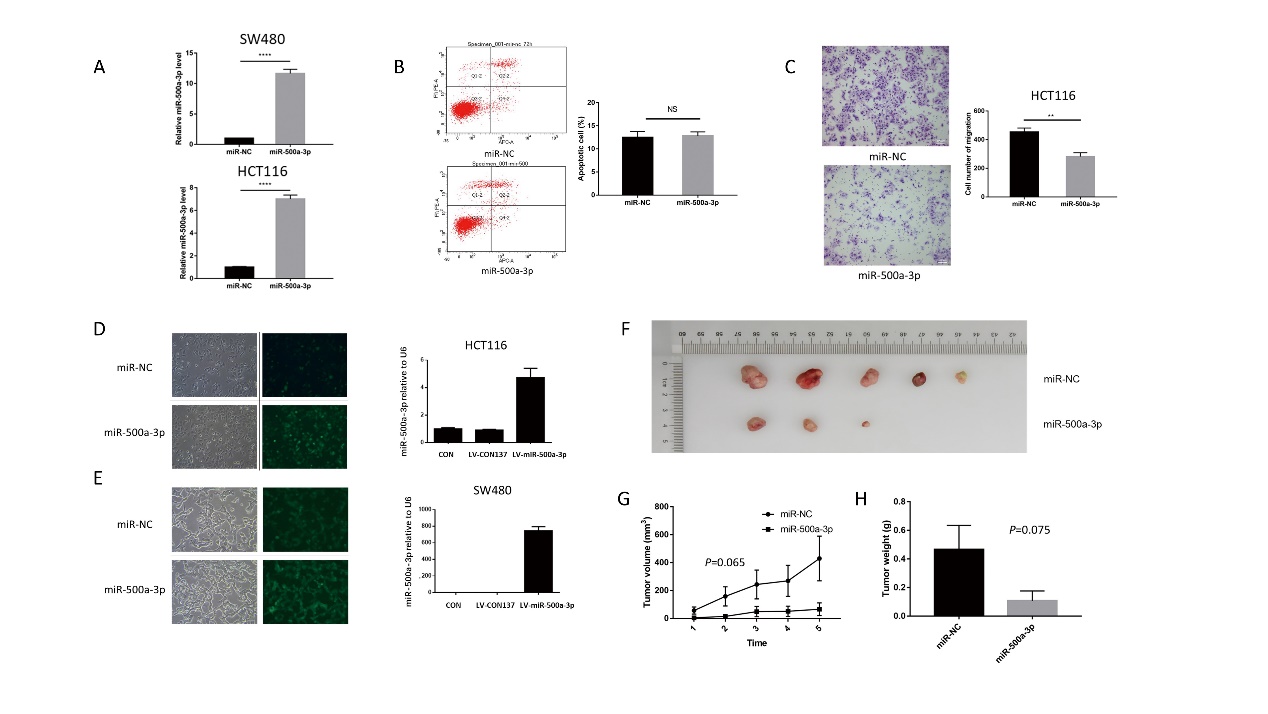
**

**Figure S2**

(A) qRT-PCR was carried out to detect the level of miR-500a-3p in SW480 and HCT116 cells transfected with miR-NC or miR-500a-3p.

(B) miR-500a-3p mimics did not increase apoptosis in HCT116 cells.

(C) miR-500a-3p mimics inhibited cell migration in HCT116 cells.

(D-E) Verification of miR-500a-3p overexpression efficiency in SW480 and HCT116 cells

(F) Representative tumor diagrams in different groups were shown.

(G-H) Tumor volume and weight in the miR-500a-3p group were significantly lower than those in the miR-NC group.

**
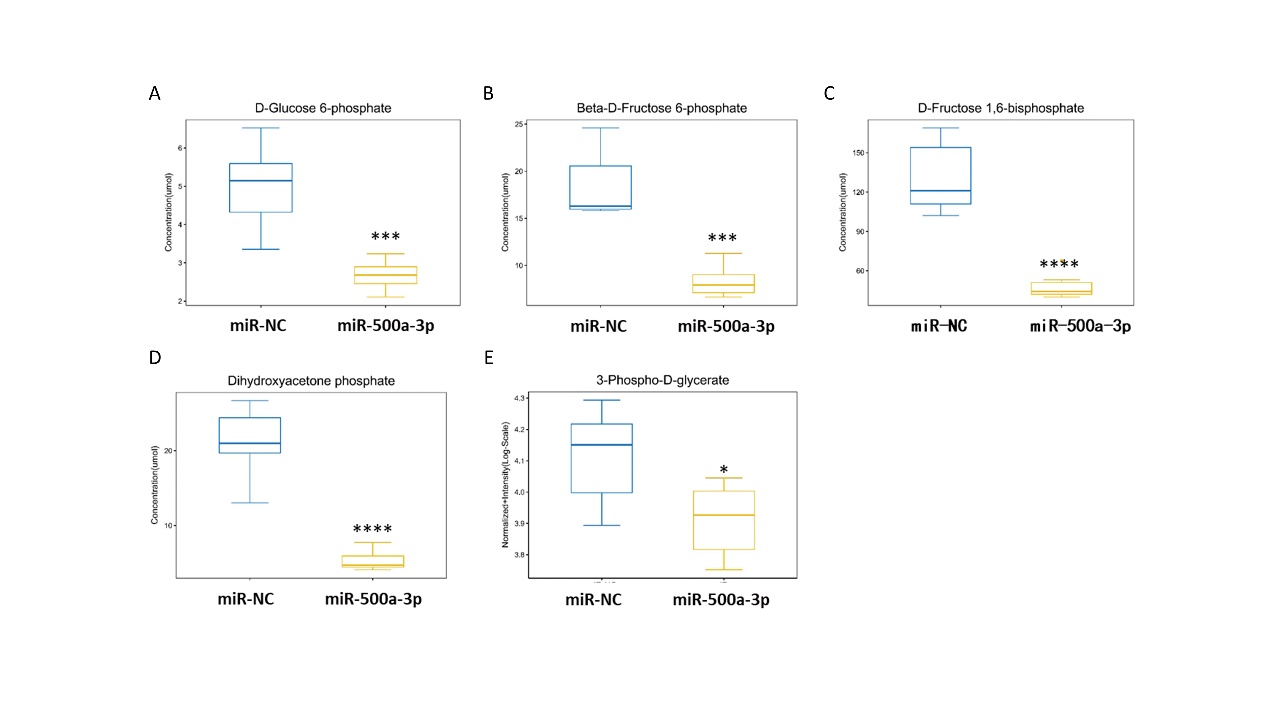
**(I) Ki-67 expression was significantly higher in tumors of miR-500a-3p group than that of miR-NC group.

**Figure S3**

(A-E) Glucose 6-phosphate (A), fructose 6-phosphate (B), fructose 1,6-bisphosphate (C), dihydroxyacetone phosphate (D) and 3-phospho-glycerate (E) in the miR-500a-3p group were significantly lower than those in the miR-NC group.

**
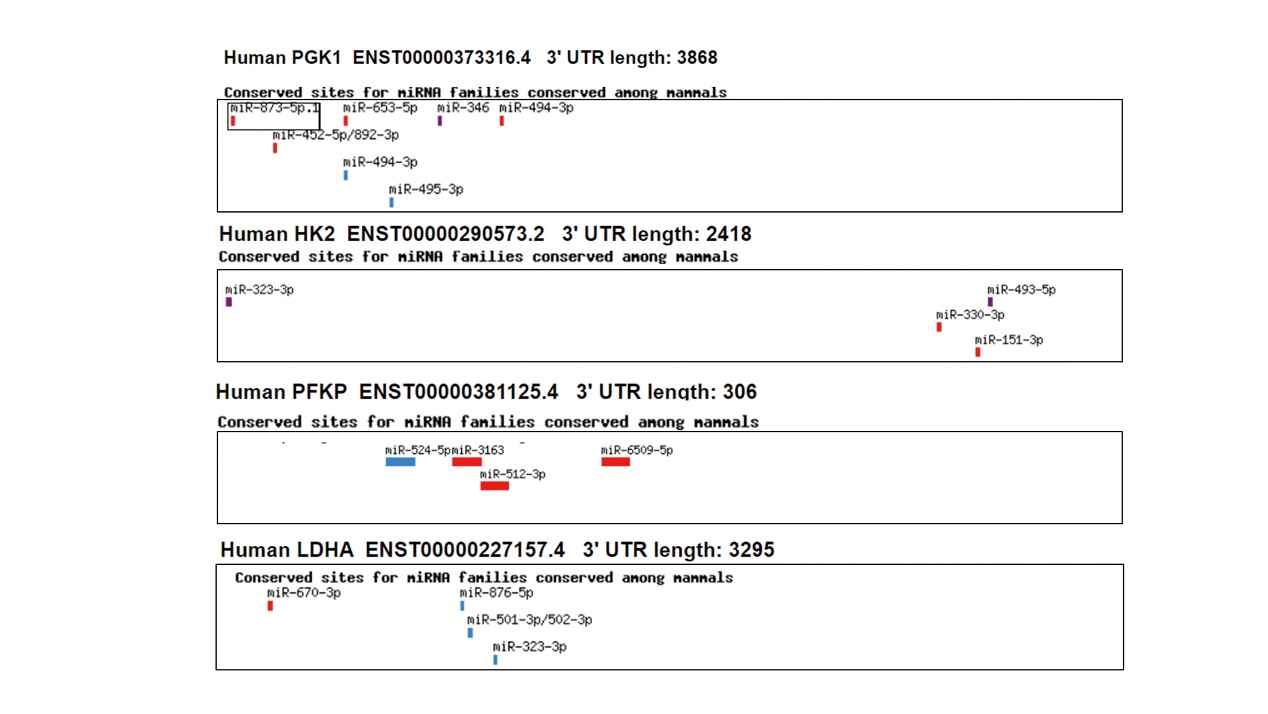
**

**Figure S4**

The 3’UTR of glycolysis enzymes did not have conserved binding site of miR-500a-3p.

**
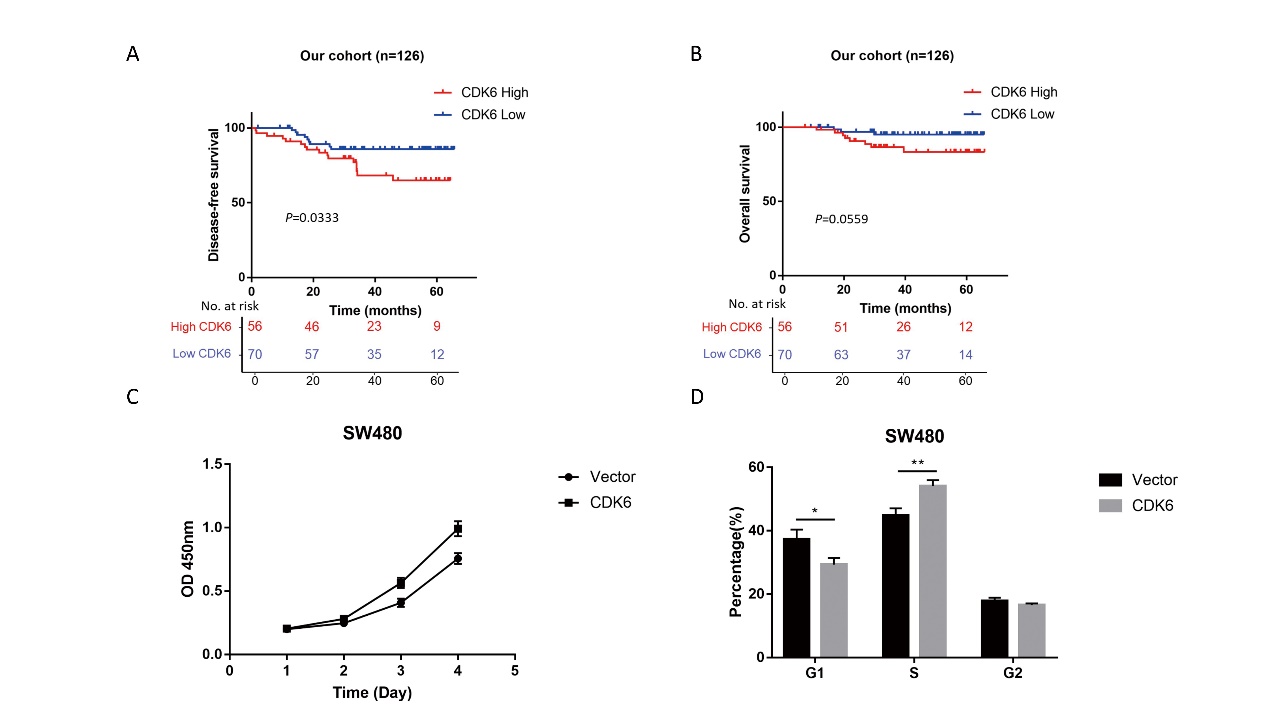
Figure S5**

(A-B) CDK6 high expression was significantly associated with poor overall survival (A) and disease-free survival (B) in CRC specimens.


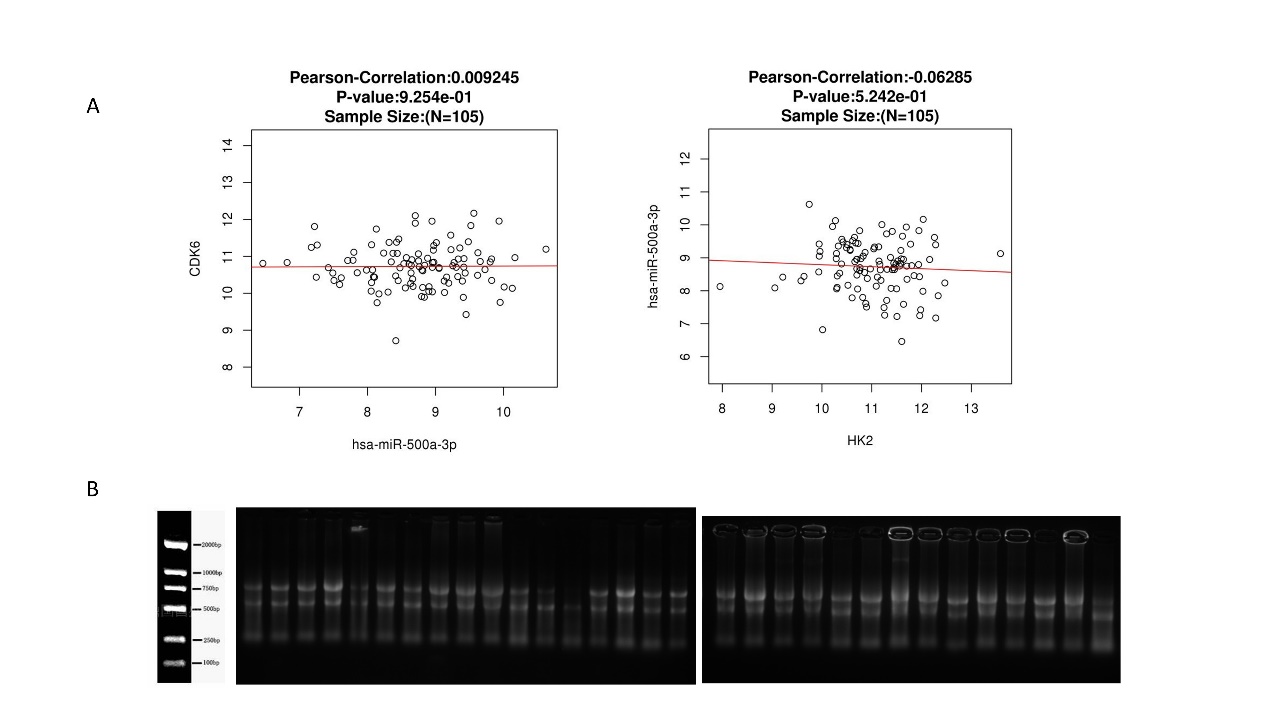
(C-D) CDK6 enhanced CRC cell proliferation using CCK-8 (C) and cell cycle assays (D). **Figure S6**

(A) The correlation between miR-500a-3p and CDK6 in CPTAC_COAD database (n=105).

(B) The correlation between miR-500a-3p and HK2 in CPTAC_COAD database (n=105).

(C) The representative images of RNA electrophoresis.
